# Protective Effects of *Curcuma longa L*. on Corticosterone-Induced Neurotoxicity and Anti-Depression-Like Behavior: Involvement of NMDA and 5-HT_7A_ Receptors

**DOI:** 10.4014/jmb.2506.06005

**Published:** 2025-08-05

**Authors:** Kyo-Nyeo Oh, Dool-Ri Oh, Youngbae Kim, Sunoh Kim

**Affiliations:** 1 Jeonnam Bio Foundation, Green Bio Headquarters, Natural Resources Laboratory, Jeollanamdo 59338, Republic of Korea; 2 Central R&D Center, B&Tech Co., Ltd., Naju 58205, Republic of Korea

**Keywords:** *Curcuma longa* L., corticosterone, anti-depressant, N-methyl-D-aspartate receptor, serotonin receptor

## Abstract

*Curcuma longa* L. is a traditional medicinal plant known for its therapeutic potential in mental disorders, including depression. Previous studies have reported its antidepressant-like effects in animal models, primarily attributed to curcumin, its major bioactive compound. However, the underlying mechanisms of these effects remain poorly understood. In this study, we investigated the neuroprotective and antidepressant-like effects of *C. longa* through the N-methyl-D-aspartate (NMDA) receptor-linked corticosterone (CORT)-induced neurotoxicity pathway in primary cultured rat cortical neurons. Among various extraction methods tested, the 80% ethanol extract of *C. longa* (CL-80) exhibited the highest neuroprotective activity and the highest concentration of curcuminoids, including curcumin. Further fractionation of the CL-80 by polarity revealed that the *n*-butanol fraction (*n*-BuOH Fr.) demonstrated the most potent neuroprotective effect, with curcumin identified as the primary active constituent. Additionally, serotonin (5-hydroxytryptamine; 5-HT) receptor screening indicated that both the CL-80 and curcumin selectively modulate the 5-HT_7A_ receptor. In a restraint stress-induced depression mouse model, CL-80 and curcumin administration significantly mitigated stress-induced behavioral changes, including the prolongation of immobility time and reduction in climbing time during the forced swim test (FST). These findings suggest that *C. longa* exerts its antidepressant-like effects via modulation of NMDA and 5-HT_7A_ receptor-mediated pathways, highlighting its potential for development as a novel antidepressant agent.

## Introduction

Psychological stress (PS) is strongly associated with an increased risk of developing depression and anxiety disorders [1]. While PS can be triggered by physical or emotional external stimuli, its origin is rooted in psychological responses and is linked to an individual's internal perception of those stimuli [2]. Depression and anxiety disorders represent two of the most prevalent public health challenges due to their resistance to treatment and their association with elevated suicide risk, placing a significant burden on both patients and society [3]. Recurrent PS activates the sympathetic–adrenal medulla (SAM) axis, increasing glucocorticoid (GC) secretion, and overstimulates the hypothalamic-pituitary-adrenal (HPA) axis, ultimately leading to hippocampal neuronal damage [4, 5]. According to the classic mechanism of GC genomic action, GCs bind to their receptors, promoting the activation and nuclear translocation of GC receptor homodimers. These receptors then function as transcription factors, either enhancing or repressing the expression of target genes via DNA binding or interactions with transcriptional co-factors [6].

In contrast, nongenomic actions of GC occur rapidly (within minutes of stimulation) without requiring gene transcription, and are characterized by fast onset kinetics [7]. These nongenomic actions enhances N-methyl-D-aspartate (NMDA) receptor-mediated neurotoxicity by increasing intracellular free calcium ([Ca^2+^]*_i_*) levels and suppressing neuroprotective signaling cascades, such as the extracellular signal-regulated kinases 1 and 2 (ERK1/2) pathways linked to NMDA receptor subunit 2A (NR2A) [8].

Glutamate has emerged as a key neurotransmitter in the pathophysiology and treatment of affective disorders. Numerous preclinical studies have identified NMDA receptor, an ionotropic glutamate receptor, as promising targets for antidepressant drug development [9]. Chronic administration of a wide range of antidepressants has been shown to downregulate NMDA receptor expression [10], and some monoamine-based antidepressants directly inhibit NMDA receptor activity [11]. Unlike conventional monoamine-based antidepressants, the NMDA receptor antagonist ketamine exerts a rapid and robust antidepressant effect in clinical and preclinical models [12].

Moreover, depression has also been shown to involve GC and serotonin (5-hydroxytryptamine; 5-HT) receptors [13, 14]. 5-HT is a critical neurotransmitter involved in modulating sensory, emotional, motor, and cognitive functions [15]. Its role in the response to stress, and in the pathogenesis and treatment of depression, is well established [16]. However, despite this well-known relationship, the precise role of 5-HT receptors in depression remains unclear due to the complexity of the 5-HT receptor system, which comprises seven major families (5-HT_1_ to 5-HT_7_) and 14 subtypes, each with diverse and partially understood functions [17].

*Curcuma longa* L. (commonly known as turmeric) is a widely used culinary and medicinal herb. Its primary bioactive compound, curcumin, belongs to the curcuminoid class and has demonstrated therapeutic efficacy in various conditions, including digestive disorders [18], inflammations [19], and several types of cancer [20-22]. Notably, *C. longa* has also shown promise in the treatment of depression. For instance, Xia *et al*. reported that ethanolic extracts of *C. longa* exhibited antidepressant-like effects in a chronic mild stress model [23, 24]. Furthermore, Li *et al*. identified curcumin as the active antidepressant component and demonstrated its regulatory effects on several 5-HT receptor subtypes, including 5-HT_1a_R, 5-HT_1b_R, and 5-HT_7_R [25].

However, despite these findings, studies on the antidepressant effects of *C. longa* are limited, and the potential mechanisms involving both GC signaling and direct 5-HT receptor modulation remain unexplored. In particular, the *in vitro* and *in vivo* mechanisms of action of *C. longa* and curcumin have not been systematically investigated.

Specifically, while previous studies have independently investigated the role of either glutamatergic excitotoxicity (via NMDA receptor overactivation) [9] or serotonergic dysregulation (involving 5-HT receptors) [13, 16] in depression, few have addressed the synergistic impact of these pathways under GC-induced stress conditions. Corticosterone (CORT) has been shown to rapidly potentiate NMDA-mediated neurotoxicity through nongenomic mechanisms, leading to oxidative stress and apoptosis [8]. In parallel, chronic elevation of GC has been reported to downregulate serotonergic signaling, including 5-HT_7A_ receptor activity, which plays a role in mood regulation and synaptic plasticity [13, 14]. Therefore, in the present study, we aimed to screen *C. longa* extracts prepared using various extraction methods to identify the optimal extract and its active constituents. We evaluated their neuroprotective potential in an *in vitro* model of CORT-induced, NMDA receptor-mediated neurotoxicity. Additionally, we investigated the regulatory effects of the extracts on specific 5-HT receptor subtypes. Finally, we assessed the antidepressant-like effects of *C. longa* extract and curcumin in a restraint stress-induced depression model *in vivo*.

## Materials and Methods

### Reagents

CORT, curcumin, poly-L-lysine, 3-isobutyl-1-methylxanthine (IBMX), 4-(3-Butoxy-4-methoxybenzyl) imidazolidin-2-one (Ro 20-1724), 3-(4,5-dimethylthiazol-2-yl)-2,5-diphenyltetrazolium (MTT), and fluoxetine hydrochloride were purchased from Sigma-Aldrich (USA). Antibodies against ERK1/2 and phospho-ERK were obtained from Cell Signaling Technology (USA), while β-actin monoclonal antibodies and horseradish peroxidase (HRP)-conjugated secondary antibodies were purchased from Santa Cruz Biotechnology Inc., (USA). NMDA, 5-HT and 5-HT_7_ antagonists were obtained from Tocris (UK).Trypsin, fetal bovine serum (FBS), Neurobasal medium, and B27 supplement were purchased from Invitrogen Inc., (USA). All reagents were of analytical grade.

### Extraction and Solvent Fractionation of *C. longa*

**Fig. 1:**
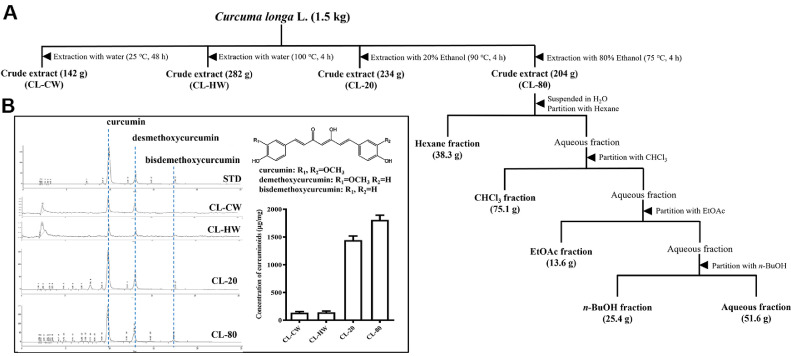
Extraction and fractionation procedure of *C. longa* and phytochemical analysis of curcuminoids. (**A**) Schematic representation of the extraction procedures for *C. longa*, including cold water (CL-CW), hot water (CL-HW), 20% ethanol (CL-20), and 80% ethanol (CL-80) extractions. The CL-80 underwent sequential solvent partitioning using *n*-hexane, chloroform (CHCl_3_), ethyl acetate (EtOAc), and *n*-butanol (*n*-BuOH), yielding five distinct fractions and the residual aqueous layer. Fraction weights are indicated. (**B**) HPLC analysis of curcuminoids in CL-CW, CL-HW, CL-20 and CL-80 and compared with standards (curcumin, demethoxycurcumin, and bisdemethoxycurcumin). Chemical structures of three major curcuminoids and their methoxy group substitutions are depicted.

*C. longa* was collected from Jindo Island, Jeollanam-do, Korea in 2022. The samples were washed, chopped into 0.5 cm pieces, and dried at 55°C for 30 h in a hot-air oven (DY-280H, Daeyoung E&B, Republic of Korea). Extraction conditions, extraction time (Ex. time, h), temperature (°C), solvent-to solid ratio (v/w, ratio), and solvent type, were varied under four conditions (Table 1). The 80% ethanol extract of *C. longa* (CL-80, 204 g) was suspended in distilled water and sequentially partitioned with *n*-hexane (3 × 1 L), chloroform (CHCl_3_; 3 × 1 L), ethyl acetate (EtOAc; 3 × 1 L), and *n*-butanol (*n*-BuOH; 3 × 1 L). The remaining aqueous phase was filtered and freeze-dried for 72 h. The extraction procedure is illustrated in Fig. 1. Extraction yield (%) was calculated as follows : Yield (%) = (M_0_ / M) × 100, where M_0_ is the dry weight of extract and M is the raw material weight [26].

### High Performance Liquid Chromatography (HPLC) and Thin-Layer Chromatography (TLC) Analysis

Samples were dissolved in 50% methanol, sonicated for 15 min, and analyzed using an Agilent 1260 HPLC system (Agilent Technologies, USA) with an Eclipse XDB-C18 column (4.6 × 250 mm, 5 μm). The mobile phase consisted of 0.1% acetic acid in water (solvent A) and 70% acetonitrile (solvent B) at a 30:70 ratio, with a flow rate of 1 ml/min and detection at 420 nm. TLC was performed on silica gel 60 F254 plates using a chloroform/methanol (48:2) solvent system. Spots were visualized by UV after spraying with 1% cerium sulfate.

### Primary Culture of Rat Cortical Neurons

Cerebral cortical neuron cultures were prepared as previously described [27]. Briefly, Cortices from day 18 (E18) Sprague-Dawley rat embryos were isolated and dissociated using 0.25% trypsin in HBSS. Cells were plated in Neurobasal medium with B27 and incubated at 37°C in 5% CO_2_. Experiments were conducted on neurons at day *in vitro* (DIV) 10.

### Neuronal Viability Assay

Neuronal viability was assessed by measuring the dehydrogenase activity retained by living cells using an MTT and lactate dehydrogenase (LDH) assays. Cultured cortical neurons were washed with Mg^2+^-free HEPES-buffered solution (in mM: 150 NaCl, 5 KCl, 2 CaCl_2_, 10 HEPES, 10 glucose, and 0.001 glycine, pH adjusted to 7.4 with NaOH) and then incubated with NMDA, CORT or both drugs conditions for various time. An MTT solution (5 mg/ml, 50 μl/well) was added to the medium and the neurons were incubated for 4 h before the supernatants were removed and the formazan crystals solubilized in 100 μl DMSO. The optical density was measured at 540 nm using a microplate reader (BioTek, USA). The cell viability was confirmed by measuring the release of the cytosolic enzyme LDH to the culture medium by using an LDH assay kit (Colorimetric; Abcam, USA). The optical density was measured at 450 nm using a microplate reader (BioTek).

### Reactive Oxygen Species (ROS) and Superoxide Dismutase (SOD) Activity Assay

ROS levels were determined using 2',7'-dichlorofluorescein diacetate (DCFH-DA) (Sigma–Aldrich) as previously reported [28]. Briefly, the cells were incubated with 2.5 μM DCFH-DA for 30 min and washed with cold PBS twice. The fluorescence at 485 nm excitation and 535 nm emission wavelength was measured using a Tecan multimode microplate reader (Tecan Trading AG, Switzerland).

SOD activity was measured using a SOD activity assay kit (BioVision, USA) according to the manufacturer’s protocol. Briefly, 20 μl of supernatant was added with 200 μl of water soluble tetrazolium working solution and 2 μl of enzyme working solution to a 96-well plate. After incubating the plate at 37°C for 20 min, the absorbance at 450 nm was read using a microplate spectrophotometer reader (BioTek).

### Western Blot Analysis

Cultured cortical neurons (10 DIV) were starved for 6 h in Neurobasal medium without any supplement as previously described [8]. Then drugs were added to the Neurobasal medium. After this treatment, lysates were prepared in SDS sample buffer and proteins separated by SDS-PAGE, transferred to PVDF membranes, and probed with anti-pERK and anti-ERK antibodies (1:1000) overnight at 4°C. The membranes were washed in TBST, incubated with the appropriate horseradish peroxidase-labeled anti-rabbit or anti-mouse IgG antibodies (1:10,000) for 1 h at room temperature and washed again. The protein signal was detected using the ECL Western Blotting Detection System (USA).

### Caspase-3 Detection

The cortical neurons were fixed, permeabilized, blocked, and incubated with anti-caspase-3 antibodies(1:100), followed by fluorescent secondary antibodies and Hoechst stain. After washing the cells, the plates were sealed and the signal measured with an ArrayScan HCS Reader (Thermo Fisher Scientific, USA).

### 5-HT Receptors-Mediated cAMP Accumulation

Human astrocytoma 1321N1 cells that stably express the human 5-HT_6_R gene (cat.ES-316-CV, clone C1, Perkin Elmer, USA) were grown in DMEM supplemented with 10% FBS, 1mM sodium pyruvate and 0.4 mg/ml geneticin (Calbiochem, Germany) at 37°C in a 95% air and 5% CO_2_ incubator. The CHO-K1 cells (ATCC CCL-61TM) were plated in complete RPMI medium on 6-well plates at a concentration of 5 × 10^5^ cells per well and then transiently transfected with human 5-HT_4_R, 5-HT_7A_R or 5-HT_7B_R for 48 h in serum free RPMI medium. After 48 h, the transfected cells were washed in the induction buffer: 1X PBS containing the phosphodiesterase inhibitors IBMX (0.5 mM) and Ro 20-1724 (0.1 mM) and the receptor agonist 5-HT (100 μM). After 15 min the reaction was terminated by adding the Parameter cAMP 1X Cell Lysis Buffer (300 μl per well) (R&D systems, USA). The fluorescence intensity of the accumulated cAMP was measured using the microplate reader (Molecular Devices, USA).

### Animals

Male ICR mice (18–20 g) and male Sprague-Dawley rats (180–200 g) were purchased from Central Lab Animal Inc., (Republic of Korea). The animals were housed at a constant room temperature of 22 ± 2°C with 50 ± 5%humidity, under a 12 h light-dark cycle (light on at 8:00 am), and given free access to water and food. The animals were allowed to acclimatize to their new environment for 4 days before the beginning of the experiments. All experimental procedures were conducted in accordance with the relevant guidelines for the care of experimental animals and approved by the institutional animal care and use committee (IACUC) of Bioresources and Technology (B&Tech) Co., Ltd., Republic of Korea (approval number BT-003-2022).

### Stress Induction

Stress was induced following a slightly modified method previously described [29]. Briefly, the mice were restrained for 2 h per day for 14 days in a 50 ml conical tube with holes for breathing. Since the restrained mice could not eat nor drink, the control group was similarly food-and water-deprived for 2 h.

### Experimental Groups and Drug Administration

A total of 70 mice were randomly assigned to 7 treatment groups of 10 mice each (N = 10 per group): non-stressed plus 1% Tween-20 (control group); stress-exposed, 1% Tween-20 (stress group); stress-exposed, 20 mg/kg body weight (b.w.) fluoxetine (Fluoxetine group); stress-exposed, 100 mg/kg b.w. CL-80 extract (100 mg/kg group); stress-exposed, 300 mg/kg b.w. CL-80 extract (300 mg/kg group); stress-exposed, 5 mg/kg b.w. curcumin (5 mg/kg group); and stress-exposed, 10 mg/kg b.w. curcumin (10 mg/kg group). CL-80 extract and curcumin were dissolved in 1% Tween-20 and orally administered once per day at 9:00 am for 15 days; stress was induced for 14 days, starting on the second day of treatment. The Fluoxetine group was injected with fluoxetine intraperitoneally (i.p.) 30 min before the induction of stress for 3 days.

### Forced Swimming Test (FST)

To confirm whether CL-80 and curcumin possess antidepressant-like activities, the mice underwent FST according to a slightly modified previously described method [30, 31], were placed individually inside glass cylinders (height = 50 cm, diameter = 15 cm) containing 30 cm deep water at a temperature of 27°C. All the mice were allowed to adapt to the water for 15 min on the day before the test. After restraint stress induction on day 15, the animals were immediately tested for 5 min. During the test session, immobility and climbing times were analyzed using a Smart ver. 2.5 video tracking system (Panlab, Spain). Climbing behavior consisted of upward-directed movements of the forepaws along the side of the swim chamber. Immobility was defined as no additional activity being observed other than the actions required to keep the animal’s head above the water.

### Statistical Analysis

All data are presented as mean ± standard deviation (SD). For *in vitro* experiments (including MTT, LDH, ROS, SOD, and caspase-3 assays), data were obtained from three independent experiments (*n* = 3 biological replicates per group), each conducted with triplicate technical replicates (*n* = 3 wells) unless otherwise stated. Behavioral data (immobility, climbing, and swimming times) were analyzed using one-way analysis of variance (ANOVA), followed by Duncan’s multiple range test for post hoc comparisons. A *p*-value less than 0.05 (*p* < 0.05) was considered statistically significant. All statistical analyses were conducted using GraphPad Prism (version 9.0).

## Results

### Quantitative Analysis of Curcuminoids in *C. longa* Extracts

To identify the optimal extraction condition for curcuminoid enrichment, *C. longa* was subjected to various extraction protocols using cold water (CL-CW), hot water (CL-HW), 20% ethanol (CL-20), and 80% ethanol (CL-80) as solvents. The extracted samples were analyzed via HPLC to quantify the contents of the three major curcuminoids: curcumin, demethoxycurcumin, and bisdemethoxycurcumin (Fig. 1).

As shown in Fig. 1B, the HPLC chromatograms revealed three distinct peaks corresponding to curcumin, demethoxycurcumin, and bisdemethoxycurcumin, which were identified based on their retention times and comparison with the standard mixture (STD). These peaks were observed at consistent retention times across all extract samples, confirming the presence of the target compounds. Among the tested conditions, the CL-80 exhibited the highest concentration of curcuminoids, followed by the CL-20, with CL-CW and CL-HW extracts showing substantially lower levels. Quantitative comparison demonstrated that the total curcuminoid content in the CL-80 reached approximately 1,808.72 ± 82.90 μg/mg, significantly higher than that in the CL-20. (1,442.44 ± 75.50 μg/mg), CL-HW (142.06 ± 21.20 μg/mg), and CL-CW (135.39 ± 19.08 μg/mg). The chemical structures of curcuminoids are shown in the inset of Fig. 1B. Curcumin (R_1_ = R_2_ = OCH_3_), demethoxycurcumin (R_1_ = OCH_3_, R_2_ = H), and bisdemethoxycurcumin (R_1_ = R_2_ = H) differ by the degree of methoxylation, which may influence both their polarity and extraction efficiency under varying solvent conditions. These results suggest that ethanol, particularly at higher concentration (80%), is a superior solvent for efficient extraction of curcuminoids from *C. longa*. This condition was therefore selected for subsequent fractionation and biological evaluation experiments.

### Neuroprotective Effects of *C. longa* Extracts against CORT- and NMDA-Induced Genomic Neurotoxicity

**Fig. 2:**
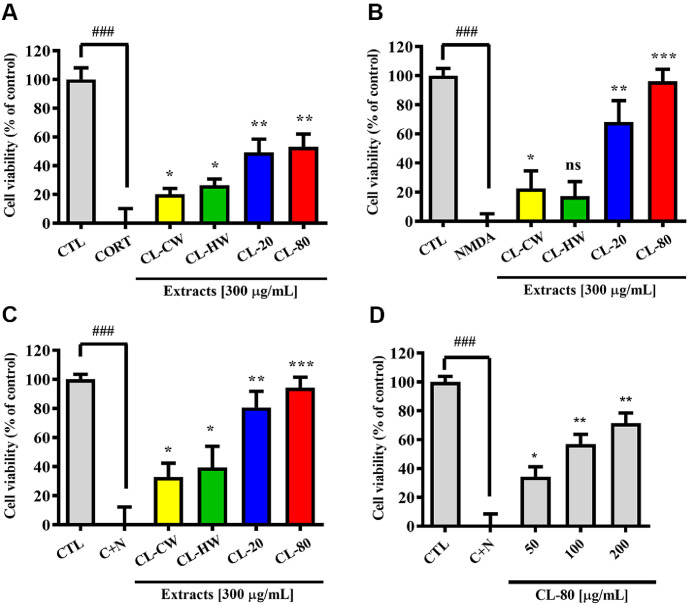
Neuroprotective effects of *C. longa* extracts against CORT- and NMDA-induced neurotoxicity in primary cortical neurons. (**A**) Genomic neurotoxicity was induced by treating primary cultured rat cortical neurons with corticosterone (CORT, 1 mM) for 24 h. Cells were pretreated for 2 h with various *C. longa* extracts, including cold water (CLCW), hot water (CL-HW), 20% ethanol (CL-20), and 80% ethanol (CL-80) extracts, followed by MTT assay to assess cell viability. (**B**) To evaluate protection against NMDA receptor-mediated delayed neurotoxicity, neurons were exposed to NMDA (100 μM) for 15 min and then incubated for 24 h. Cell viability was measured using the MTT assay. (**C**) A nongenomic neurotoxicity model was established by co-treating neurons with low-dose CORT (100 nM) and NMDA (50 μM) for 15 min, mimicking rapid stress-amplified excitotoxic conditions. (**D**) Neurons were pretreated with increasing concentrations of CL- 80 (50, 100, and 200 μg/ml), resulting in a dose-dependent restoration of cell viability under CORT + NMDA-induced nongenomic toxicity. All data are expressed as mean ± S.D. Statistical analysis was performed using unpaired Student’s *t*-test. *p < 0.05, **p < 0.01, ***p < 0.001 compared with the corresponding CORT-, NMDA or CORT+NMDA (C+N)-treated groups. ^###^*p* < 0.001 indicates a significant difference compared to the untreated control group (CTL).

To investigate the neuroprotective efficacy of *C. longa* extracts against both genomic and nongenomic pathways of neurotoxicity, we established an *in vitro* cortical neuron model in which neurodegeneration was induced by CORT, NMDA, or their combination (Fig. 2). To investigate the genomic neurotoxic effects of chronic glucocorticoid exposure, primary cortical neurons were treated with corticosterone (1 mM) for 24 h. As shown in Fig. 2A, among the tested conditions, the CL-80 showed the most pronounced protective effects (53.22 ± 8.81%, *p* <0.01). The CL-20 also showed moderate protection (49.40 ± 9.10%, *p* < 0.01), while CL-CW (20.11 ± 4.18%, *p* <0.05) and CL-HW (26.48 ± 4.23%, *p* < 0.05) extracts demonstrated minimal effects. These results suggest that *C. longa* extracts, particularly those enriched in curcuminoids, may modulate GR-dependent transcriptional cascades involved in stress-related neuronal damage. To assess whether *C. longa* extracts could also protect against excitotoxic injury induced by glutamatergic stimulation, cortical neurons were exposed to NMDA (100 μM) for 15 min and then incubated for 24 h to evaluate delayed neurotoxicity (Fig. 2B). This model represents a rapid-onset but transcription dependent (genomic) mechanism, wherein NMDA receptor activation triggers downstream gene regulatory cascades contributing to neuronal injury [32]. Therefore, Fig. 2B illustrates the response of cortical neurons to NMDA-induced excitotoxicity, which mimics genomic neurotoxicity via overactivation of NMDA receptors. Pretreatment with CL-80 significantly attenuated neuronal death (96.28 ± 8.12%, *p* < 0.001) indicating a potent protective effect against NMDA-mediated excitotoxicity.

### Neuroprotective Effects of *C. longa* Extracts against CORT- and NMDA-Induced Nongenomic Neurotoxicity

Xiao *et al*. reported a rapid, nongenomic CORT action that enhances NMDA neurotoxicity in hippocampal neurons by facilitating NMDA-induced [Ca^2+^]*_i_* increment and attenuating the NR2A-ERK1/2-mediated neuroprotective signaling [8]. To evaluate the neuroprotective efficacy of *C. longa* extracts under such nongenomic stress conditions, we examined their effects against CORT-sensitized NMDA neurotoxicity in primary cultured rat cortical neurons using the MTT assay (Fig. 2C). Exposure to low concentrations of CORT (100 nM) or NMDA (50 μM) for 15 min did not significantly affect neuronal viability when assessed 24 h later (*p* > 0.05, data not shown). However, co-treatment with both CORT (100 nM) and NMDA (50 μM) markedly decreased the cell viability compared to the untreated control group. Interestingly, pretreatment with the CL-80 significantly inhibited this cytotoxicity (94.31 ± 7.14%, *p* < 0.001). CL-20 showed moderate protective activity (80.63 ± 11.09%, *p* < 0.01) whereas CL-HW (32.80 ± 9.54%, *p* < 0.05) and CL-CW (39.57 ± 14.34%, *p* < 0.05) extracts had negligible effects. As shown in Fig. 2D, pretreatment with CL-80 restored cell viability in a concentration-dependent manner. Specifically, increasing concentrations of CL-80 (50, 100, and 200 μg/ml) led to progressive improvements in neuronal survival. These data support that CL-80 exerts neuroprotective effects through both genomic and nongenomic mechanisms. These findings suggest that that CL-80 protects against acute excitotoxicity amplified by stress by modulating multiple neurodegenerative pathways.

### Mechanisms of CL-80-Mediated Neuroprotection in CORT- and NMDA-Induced Nongenomic Neurotoxicity

To further elucidate the mechanisms underlying the neuroprotective effects of CL-80, we assessed its impact on oxidative stress, antioxidant enzyme activity, and apoptosis-related protein expression in cortical neurons subjected to CORT- and NMDA-mediated nongenomic neurotoxicity.

**Fig. 3:**
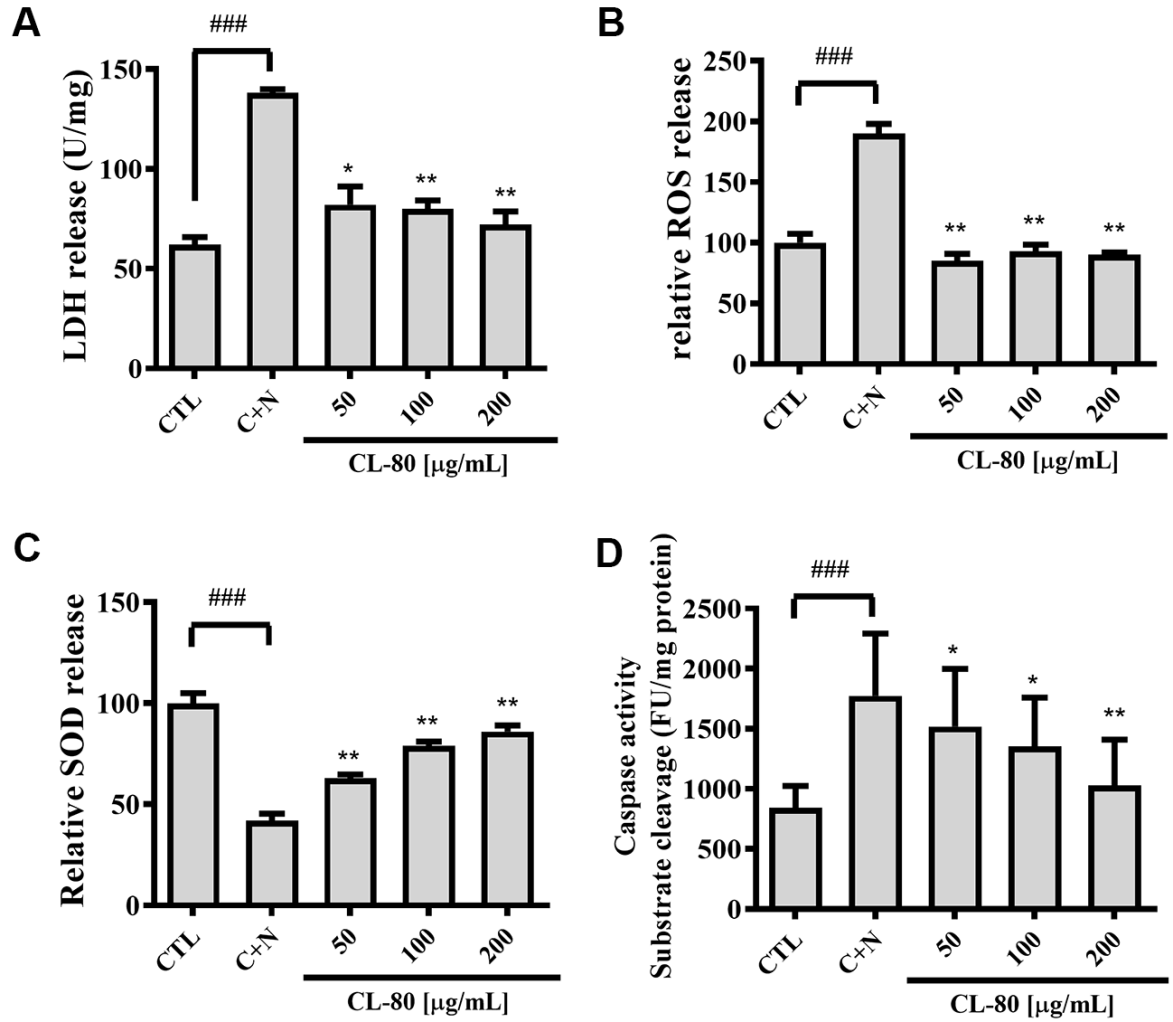
Mechanistic evaluation of CL-80-mediated neuroprotection against CORT- and NMDA-induced nongenomic toxicity in primary cortical neurons. (**A**) Lactate dehydrogenase (LDH) release was measured as a marker of neuronal membrane damage following co-treatment with corticosterone (CORT, 100 nM) and NMDA (50 μM) for 15 min. Neurons were pretreated for 2 h with CL-80 at concentrations of 50, 100, or 200 μg/ml. (**B**) Intracellular reactive oxygen species (ROS) levels were significantly elevated by CORT + NMDA treatment and were markedly reduced by CL-80 pretreatment in a concentration-dependent manner. (**C**) Superoxide dismutase (SOD) activity was decreased under CORT + NMDA conditions, but significantly restored by CL-80 pretreatment, reflecting recovery of antioxidant defense mechanisms. (**D**) Cleaved caspase- 3 expression was evaluated by immunofluorescence in primary cortical neurons exposed to CORT (100 nM) and NMDA (50 μM) for 15 min. Cells were pretreated with CL-80 (50, 100, or 200 μg/ml) for 2 h prior to exposure. Fluorescence intensity was quantified using an ArrayScan HCS Reader. All data are expressed as mean ± S.D. Statistical analysis was performed using unpaired Student’s *t*-test. *p < 0.05, **p < 0.01 compared with the corresponding CORT+NMDA (C+N)-treated groups. ^###^*p* < 0.001 indicates a significant difference compared to the untreated control group (CTL).

As shown in Fig. 3A, CORT + NMDA co-treatment resulted in a significant increase in LDH release compared to the control group (138.01 ± 1.91 *p* < 0.001). Pretreatment with CL-80 attenuated LDH release in a dose-dependent manner. The 50 and 100 μg/ml groups showed moderate reductions in LDH levels, while the 200 μg/ml group demonstrated the most pronounced suppression (72.01 ± 6.74, *p* < 0.01) suggesting robust cytoprotective effects. As shown in Fig. 3B, ROS levels were significantly elevated in the CORT + NMDA-treated group compared to the control (190.03 ± 8.08, *p* < 0.001), indicating strong oxidative insult. Notably, 200 μg/ml of CL-80 substantially suppressed ROS levels (90.03 ± 1.94, *p* < 0.01), suggesting potent inhibition of oxidative stress. As presented in Fig. 3C, SOD activity was markedly decreased following CORT + NMDA exposure (42.03 ± 3.48, *p* < 0.001), reflecting impairment of the cellular antioxidant defense system. Treatment with CL-80 restored SOD activity in a concentration-dependent manner. The 200 μg/ml group exhibited the highest SOD activity (86.03 ± 3.11, *p* <0.01), indicating restoration of enzymatic antioxidant defense. As shown in Fig. 3D, cleaved caspase-3 levels were markedly elevated following CORT (100 nM) and NMDA (50 μM) treatment (1774.05 ± 519.60, *p* < 0.001), indicating activation of apoptosis in cortical neurons. CL-80 pretreatment led to a dose-dependent reduction in cleaved caspase-3 expression.

### Biological Activities of CL-80 Solvent Fractions

**Fig. 4:**
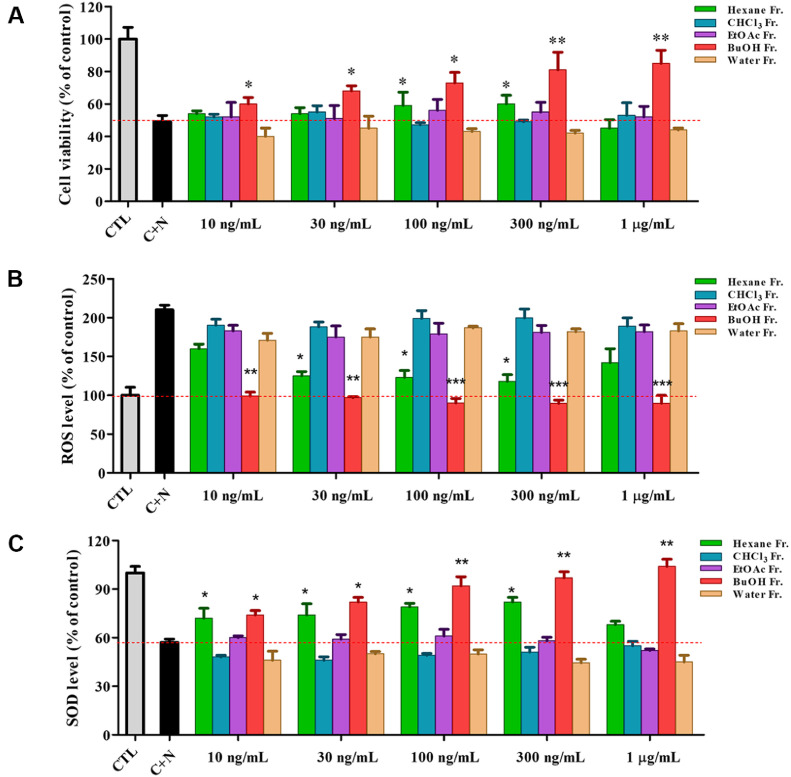
Comparative evaluation of neuroprotective effects of CL-80 solvent fractions in CORT- and NMDAinduced nongenomic neurotoxicity model. (**A**) Cell viability was assessed by MTT assay in primary cortical neurons pretreated with different solvent fractions of CL-80, *n*-hexane, chloroform (CHCl_3_), ethyl acetate (EtOAc), *n*-butanol (n- BuOH), and water, for 2 h, followed by 15-min exposure to CORT (100 nM) and NMDA (50 μM). (**B**) Intracellular reactive oxygen species (ROS) levels were markedly elevated under CORT + NMDA treatment, and were most effectively suppressed by the *n*-BuOH fraction, indicating strong antioxidative activity. (**C**) Superoxide dismutase (SOD) activity, which was reduced by CORT + NMDA co-treatment, was significantly recovered in the *n*-BuOH-treated group, further supporting its antioxidant and neuroprotective efficacy. All data are expressed as mean ± S.D. Statistical analysis was performed using unpaired Student’s *t*-test. *p < 0.05, **p < 0.01 compared with the corresponding CORT+NMDA (C+N)-treated groups. ^###^*p* < 0.001 indicates a significant difference compared to the untreated control group (CTL).

To identify the most bioactive fraction of CL-80, we subjected the CL-80 to successive solvent partitioning using *n*-hexane, chloroform (CHCl_3_), ethyl acetate (EtOAc), *n*-butanol (*n*-BuOH), and water. Each fraction was evaluated for its neuroprotective potential using three key parameters: cell viability, oxidative stress (ROS), and antioxidant enzyme activity (SOD) in CORT + NMDA-treated primary cortical neurons. As shown in Fig. 4A, treatment with *n*-BuOH fractions significantly improved neuronal survival compared to the CORT + NMDA group. Figure 4B demonstrates that the *n*-BuOH fraction consistently exhibited the most potent ROS-scavenging activity. In contrast, the EtOAc, CHCl_3_ and water fractions showed no significant activity. SOD activity was also most effectively recovered in the *n*-BuOH fraction, followed by the hexane fraction at lower concentrations (Fig. 4C). *n*-BuOH fraction significantly increased SOD levels compared to the CORT + NMDA-treated primary cortical neurons, indicating that they can activate endogenous antioxidant enzyme systems. The other fractions did not exhibit notable effects on SOD activity. Collectively, these results suggest that the neuroprotective effects of CL-80 are predominantly localized in its *n*-BuOH fraction. This fraction enhance cell survival and reduce oxidative stress by scavenging ROS and upregulating antioxidant enzymes such as SOD.

### Identification of Curcuminoids in *n*-BuOH Fraction and Validation of Curcumin’s Neuroprotective Activity

**Fig. 5:**
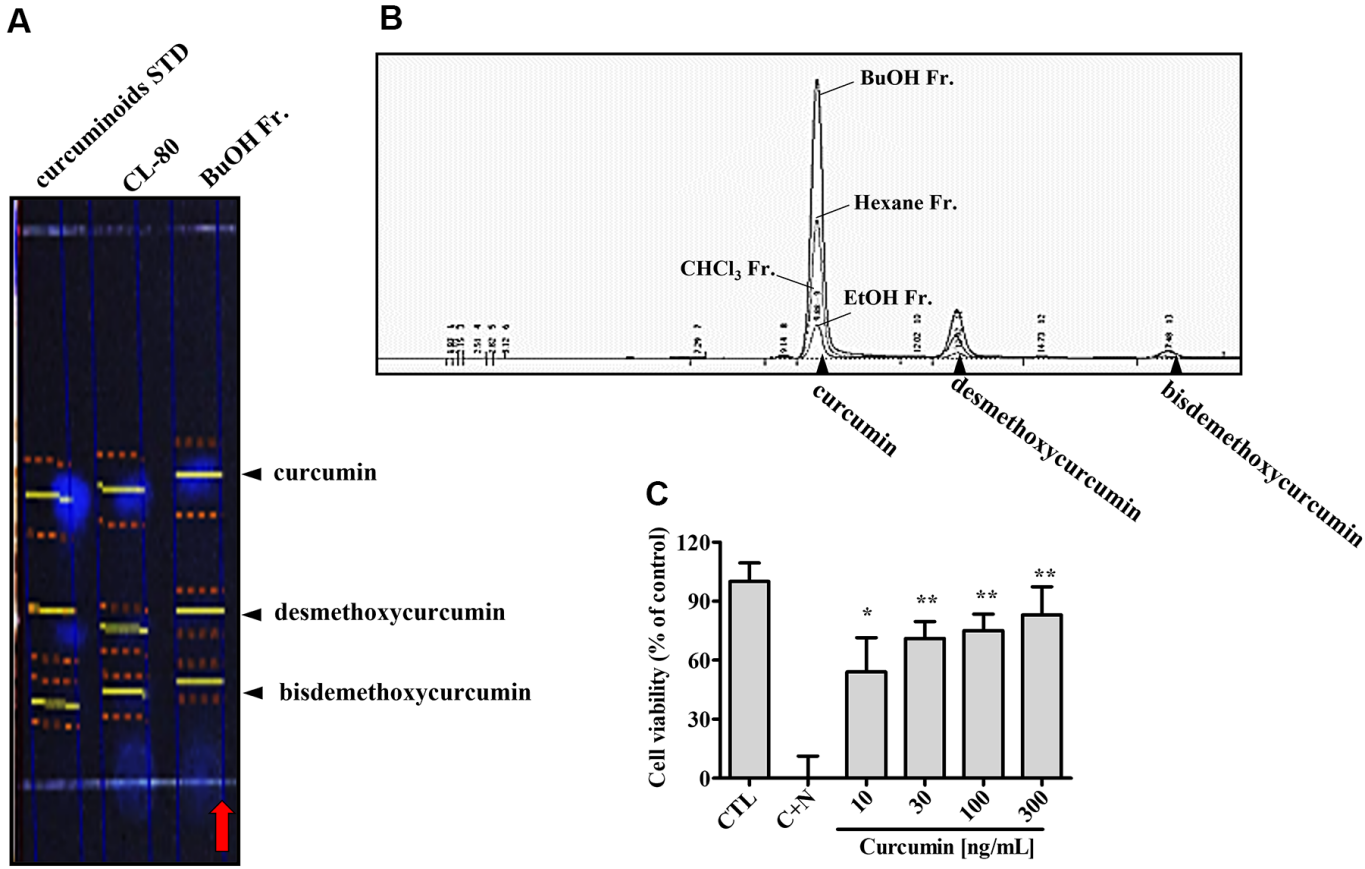
Identification of curcuminoids in the *n*-BuOH fraction and validation of curcumin as a major neuroprotective constituent. (**A**) Thin-layer chromatography (TLC) analysis of the *n*-BuOH fraction revealed three major fluorescent bands corresponding to curcumin, demethoxycurcumin, and bisdemethoxycurcumin, which were identified by co-migration with authentic standards under UV light. (**B**) High-performance liquid chromatography (HPLC) analysis confirmed that the *n*-BuOH fraction contains high levels of curcuminoids, with curcumin as the predominant component. Chromatographic profiles of the *n*-BuOH fraction were compared with standard curcuminoid mixtures. (**C**) To verify the neuroprotective activity of curcumin, primary cortical neurons were exposed to CORT (100 nM) and NMDA (50 μM) for 15 min following a 2-h pretreatment with curcumin at various concentrations. Cell viability was assessed by MTT assay, and curcumin demonstrated a dose-dependent protective effect against neurotoxicity. All data are expressed as mean ± S.D. Statistical analysis was performed using unpaired Student’s *t*-test. *p < 0.05, **p < 0.01 compared with the corresponding CORT+NMDA (C+N)-treated groups.

To identify the bioactive constituents responsible for the observed neuroprotective effects of the *n*-BuOH fraction of CL-80, we performed phytochemical analysis using TLC and HPLC. As shown in Fig. 5A, TLC analysis revealed three major fluorescent bands in the *n*-BuOH fraction, corresponding to curcumin, demethoxycurcumin, and bisdemethoxycurcumin, as verified by co-migration with standard compounds under UV light. These results indicated the presence of curcuminoids as major constituents in the *n*-BuOH fraction. Subsequent HPLC profiling further confirmed the high curcuminoid content in the *n*-BuOH fraction (Fig. 5B). The retention times and peak areas of the *n*-BuOH sample closely matched those of authentic standards for curcumin, demethoxycurcumin, and bisdemethoxycurcumin, with curcumin being the predominant component. Other solvent fractions, including EtOH, CHCl_3_, and hexane, exhibited significantly lower curcuminoid content, highlighting *n*-BuOH as the most enriched fraction. To validate the functional contribution of curcumin, we examined its neuroprotective activity in primary cortical neurons exposed to CORT (100 nM) and NMDA (50 μM), a model of nongenomic neurotoxicity. As shown in Fig. 5C, curcumin treatment dose-dependently improved cell viability. This indicates that curcumin is a major bioactive component contributing to the neuroprotective efficacy of the *n*-BuOH fraction. These findings demonstrate that the *n*-BuOH fraction of CL-80 contains high levels of curcuminoids, with curcumin as the dominant active ingredient. The potent neuroprotective effect of curcumin underlines its mechanistic role in mitigating stress-induced neuronal injury, supporting its utility as a key marker compound for standardization and therapeutic development.

### CL-80 Specifically Inhibits 5-HT_7A_ Receptor

**Fig. 6:**
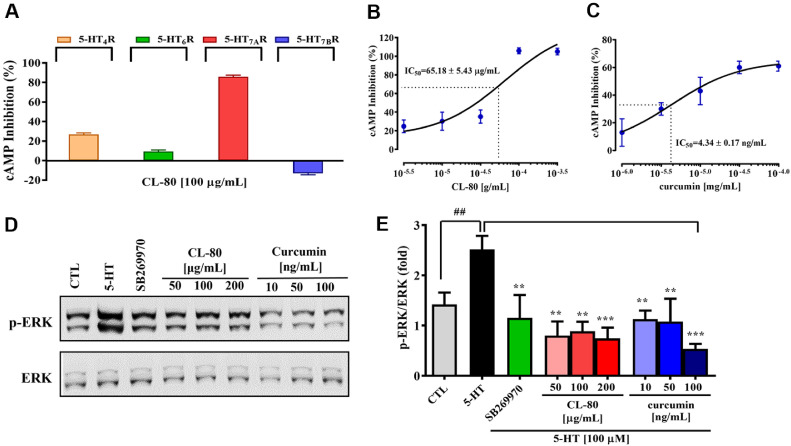
Inhibitory effects of CL-80 and curcumin on 5-HT_7A_ receptor-mediated cAMP signaling and ERK1/2 phosphorylation. (**A**) CHO-K1 cells overexpressing human 5-HT receptor subtypes (5-HT_4_R, 5-HT_7A_R, and 5-HT_7B_R), as well as human astrocytoma 1321N1 cells expressing 5-HT_6_R, were pretreated with CL-80 (100 μg/ml) for 15 min before stimulation with 5-HT (100 μM). (**B**) Dose-dependent inhibition of cAMP accumulation was observed in 5-HT_7A_Roverexpressing CHO-K1 cells following pretreatment with increasing concentrations of CL-80 (3–300 μg/ml). (**C**) Curcumin (1–100 ng/ml)treatment also dose-dependently suppressed 5-HT-induced cAMP levels in 5-HT_7A_R-overexpressing cells, comparable to the effects of CL-80. (**D, E**) Western blot analysis showed that 5-HT stimulation significantly increased ERK1/2 phosphorylation, which was effectively attenuated by SB-269970 (a selective 5-HT_7A_R antagonist), CL-80, and curcumin. All data are expressed as mean ± S.D. Statistical analysis was performed using unpaired Student’s *t*-test. *p < 0.05, **p < 0.01, ***p < 0.001 compared with the corresponding 5-HT-treated groups. ^###^*p* < 0.001 indicates a significant difference compared to the untreated control group (CTL).

Previous studies have demonstrated that chronic stress or CORT-induced downregulation of 5-HT_1_A, 5-HT_2_, and 5-HT_4_ receptor mRNA levels contributes to the antidepressant-like effects of curcumin [33-35]. However, the direct modulation of 5-HT receptor subtypes by *C. longa* and curcumin in relation to neuronal plasticity has not been previously characterized. To elucidate the receptor subtype-specific modulatory effects of CL-80 on serotonergic signaling, we measured intracellular cyclic AMP (cAMP) levels in various 5-HT receptor-overexpressing cell lines following CL-80 treatment (Fig. 6). CHO-K1 cells overexpressing human 5-HT_4_R, 5-HT_7A_R, or 5-HT_7B_R, and human astrocytoma 1321N1 cells stably expressing human 5-HT_6_R, were utilized to examine subtype-selective responses. Treatment with CL-80 alone did not significantly alter cAMP levels in any of the 5-HT receptor overexpressing cell lines (data not shown). As shown in Fig. 6A, treatment with 5-HT (10 μM), a non-selective serotonin receptor agonist, markedly elevated intracellular cAMP levels in all tested cell lines (data not shown). However, pretreatment with CL-80 (100 μg/ml, 15 min) resulted in a significant and selective inhibition of cAMP accumulation exclusively in 5-HT_7A_R-overexpressing cells (85.83 ± 1.71%). Notably, CL-80 did not significantly affect 5-HT-induced cAMP levels in cells overexpressing 5-HT_4_R, 5-HT_7B_R, or 5-HT_6_R. A dose-dependent study further confirmed the specificity and potency of CL-80 in modulating 5-HT_7A_R signaling. As shown in Fig. 6B, increasing concentrations of CL-80 (3, 10, 30, 100 and 300 μg/ml) progressively reduced intracellular cAMP levels in 5-HT_7A_R-overexpressing cells following 5-HT stimulation, demonstrating a clear dose-response relationship (IC_50_ = 65.18 ± 5.43 μg/ml). These findings strongly suggest that CL-80 selectively inhibits 5-HT_7A_R-mediated signaling pathways. To determine whether curcumin, the principal active compound in CL-80, exhibits a similar receptor-specific modulatory profile, we tested its effect in the same model system. As shown in Fig. 6C, pretreatment with curcumin (1, 3, 10, 30, and 100 ng/ml) also significantly suppressed 5-HT-induced cAMP accumulation in 5-HT_7A_R-overexpressing cells (IC_50_ = 4.34 ± 0.17 ng/ml), comparable to the effect observed with CL-80. This result suggests that curcumin contributes to the functional activity of CL-80.

To further investigate downstream signaling, we evaluated ERK1/2 phosphorylation in 5-HT_7A_R-overexpressing cells. As shown in Fig. 6D and 6E, 5-HT stimulation significantly increased ERK1/2 phosphorylation relative to control. However, pretreatment with either SB-269970, a selective 5-HT_7A_R antagonist, or CL-80 and curcumin effectively attenuated 5-HT-induced ERK1/2 phosphorylation. Taken together, these findings demonstrate that CL-80 exerts selective antagonist-like effects on 5-HT_7A_R, without impacting other serotonergic receptor subtypes. This receptor selectivity may underlie the neuroprotective and antidepressant-like properties attributed to CL-80 and its active constituent, curcumin.

### Antidepressant-Like Effects of CL-80 and Curcumin in a Restraint Stress-Induced Mouse Model

**Fig. 7:**
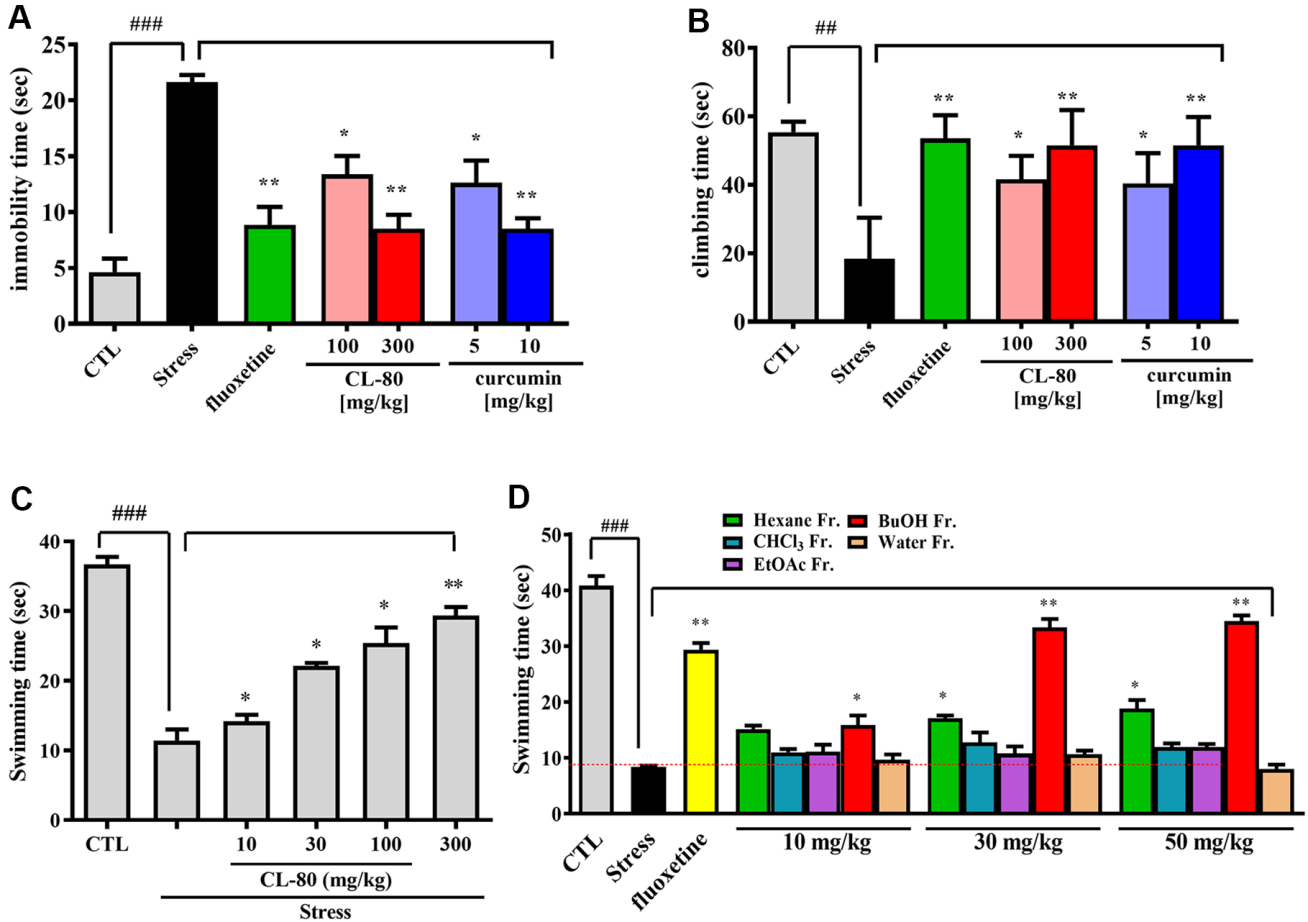
Antidepressant-like effects of CL-80, curcumin, and solvent fractions in a mouse restraint stressinduced depression model assessed via the forced swim test (FST). (**A**) Immobility time was significantly increased in the stress-exposed group compared to the control group, indicating depression-like behavior. Treatment with CL-80 (100 and 300 mg/kg) and curcumin (5 and 10 mg/kg) significantly reduced immobility time, comparable to the fluoxetine (20 mg/ kg, i.p.) positive control. (**B**) Climbing time, a behavioral marker of noradrenergic activity, was markedly reduced by stress but restored by CL-80 and curcumin in a dose-dependent manner. (**C**) Swimming time, considered indicative of serotonergic antidepressant activity, was significantly increased by CL-80 treatment compared to the stress group. (**D**) To identify the active fraction contributing to antidepressant-like effects, solvent fractions of CL-80 (*n*-hexane, CHCl_3_, EtOAc, *n*-BuOH, and water) were administered (10–50 mg/kg). Among these, the *n*-BuOH fraction significantly increased swimming time in a dosedependent manner, with effects comparable to fluoxetine. Data are expressed as mean ± standard deviation (S.D.) from 10 mice per group. Statistical significance was determined using one-way ANOVA followed by post hoc analysis. *p < 0.05, **p < 0.01, and ***p < 0.001 indicate significant differences compared to the stress-only group. ^##^*p* < 0.01 and ^###^*p* < 0.001 indicates significant difference compared to the non-stressed control group.

To assess the antidepressant-like properties of CL-80 and its active component curcumin, a restraint stress-induced depression model was employed using mice. Mice were orally administered with either CL-80 (100 or 300 mg/kg), curcumin (5 or 10 mg/kg), fractions of CL-80 (10, 30 and 50 mg/kg) or fluoxetine (positive control, 20 mg/kg) once daily for 14 consecutive days, followed by behavioral assessment using the forced swim test (FST) (Fig. 7).

Mice exposed to restraint stress exhibited a significant increase in immobility time (21.63 ± 0.65 sec), compared with the non-stressed control group (4.62 ± 1.20 sec; *p* < 0.001), indicating depression-like behavior (Fig. 7A). However, pretreatment with CL-80 at 100 and 500 mg/kg significantly reduced the immobility time to 13.38 ± 1.65 sec (*p* < 0.05 vs. stress group) and 8.50 ± 1.25 sec (*p* < 0.01 vs. stress group), respectively. Similarly, curcumin-treated groups (5 and 10 mg/kg) showed reduced immobility times of 12.63 ± 1.98 sec (*p* < 0.05 vs. stress group) and 8.50 ± 0.94 sec, respectively (*p* < 0.01), indicating a reversal of stress-induced depressive behavior.

In addition, climbing time, a behavioral indicator of active coping, was markedly reduced in the stress group (18.37 ± 11.99 sec, *p* < 0.01) compared with the control group (55.37 ± 3.02 sec) (Fig. 7B). Treatment with CL-80 (100 and 300 mg/kg) or curcumin (5 and 10 mg/kg) significantly restored climbing behavior (41.63 ± 6.82, 51.5 ± 10.32, 40.4 ± 8.84, and 51.5 ± 8.26 sec, respectively), further supporting the antidepressant-like potential of these compounds.

Furthermore, swimming time, a behavioral indicator associated with serotonergic activity, was markedly affected. As shown in Fig. 7C, the stress group exhibited significantly reduced swimming time (11.43 ± 1.59 sec, *p* < 0.001) compared to the control group (36.73 ± 1.06 sec). However, treatment with CL-80 significantly increased swimming time in a dose-dependent manner. Specifically, to determine which solvent fraction of CL-80 contributes to its antidepressant-like activity, we evaluated the behavioral effects of hexane, chloroform (CHCl_3_), ethyl acetate (EtOAc), *n*-butanol (BuOH), and water fractions at doses of 10, 30, and 50 mg/kg in the FST (Fig. 7D). Among all fractions, the *n*-BuOH fraction exhibited a dose-dependent and statistically significant increase in swimming time compared to the stress group. Notably, administration of the *n*-BuOH fraction at 30 mg/kg and 50 mg/kg resulted in swimming times of approximately 33–35 sec, which were comparable to or exceeded that of the fluoxetine-treated group (29.34 ± 1.23 sec). In contrast, the other fractions (*n*-hexane, CHCl_3_, EtOAc, and water) showed only marginal or non-significant effects at all tested doses, with swimming times remaining below 20 sec. These findings strongly suggest that the *n*-BuOH fraction contains the principal active constituents responsible for the antidepressant-like effects of CL-80.

## Discussion

Current antidepressant therapies primarily include selective serotonin reuptake inhibitors (SSRIs), norepinephrine reuptake inhibitors (NRIs), monoamine oxidase (MAO) inhibitors, and tricyclic antidepressants. Although these agents are widely prescribed, their therapeutic efficacy is often limited, and their use is frequently associated with undesirable side effects such as cardiotoxicity, hypertension, sexual dysfunction, weight gain, and sleep disturbances [36-38]. Given these limitations, there is a growing demand for safer, multifaceted antidepressant agents that address both emotional and somatic symptoms of depression. Accordingly, recent research has increasingly focused on identifying natural products with improved tolerability and fewer side effects [38, 39].

Using *in vitro* assays, we screened various natural compounds for their protective effects against psychological stress (PS)-induced cytotoxic mechanisms. Among the compounds tested (data not shown), *C. longa* emerged as the most promising candidate, exhibiting the strongest cytoprotective activity. Based on these findings, we focused on this widely used traditional medicinal plant and evaluated its antidepressant-like potential.

In this study, we demonstrated that *C. longa* extracts exhibit potent neuroprotective and antidepressant-like effects via both genomic and nongenomic mechanisms in CORT-induced neurotoxicity models. Among the tested preparations, the CL-80 showed the most pronounced neuroprotective activity and was subsequently subjected to solvent fractionation. The *n*-BuOH fraction of CL-80 was identified as the most bioactive, enriched with curcuminoids, particularly curcumin, as confirmed by HPLC and TLC analyses.

Chronic PS and elevated GC levels contribute to neurodegeneration and depression, primarily through NMDA receptor overactivation leading to intracellular Ca^2+^ influx, oxidative stress, and apoptosis [4, 8, 9]. Our *in vitro* results indicate that CL-80 effectively attenuates both genomic and nongenomic neurotoxicity, in part by downregulating caspase-3 activation, a key effector of apoptosis and neuronal plasticity [40, 41].

CORT is known to exert both genomic and nongenomic effects in the central nervous system [42, 43]. In this study, we screened various *C. longa* extracts for their neuroprotective effects against CORT-induced genomic neurotoxicity in a cell model. In our cell model, CL-80 restored cell viability following 24 h of high-concentration CORT exposure, indicating its genomic neuroprotective effect.

Notably, CORT has been reported to rapidly potentiate NMDA-mediated neurotoxicity through nongenomic actions by elevating intracellular calcium levels and suppressing NR2A-ERK1/2 protective signaling [8]. However, *C. longa* and curcumin effects on CORT nongenomic mechanisms have not been fully evaluated *in vitro* or *in vivo*. Therefore, we investigated *C. longa* neuroprotection against CORT-induced, NMDA-mediated nongenomic neurotoxicity in primary cultures of rat cortical neurons with MTT assays (Fig. 4). Interestingly, our findings reveal that low-dose CORT and NMDA co-treatment rapidly reduced neuronal viability within 15 min, consistent with a nongenomic mechanism. Moreover, this nongenomic neurotoxicity could be blocked by CL-80 in a dose-dependent manner. However, the mechanisms of CORT-induced nongenomic neurotoxicity are still unknown. Therefore, to elucidate the protective mechanisms of *C. longa* against CORT-NMDA-induced nongenomic toxicity, we assessed caspase-3 activity. Caspase-3, a key executioner of apoptosis, was significantly upregulated under co-treatment conditions but was markedly suppressed by CL-80. These results support the role of CL-80 in inhibiting mitochondria-mediated apoptotic cascades [44, 45]. Several studies indicate that caspase-3 activation may participate in the apoptotic cell death and synaptic dysfunction observed in depression [46]. CORT-NMDA co-treatment also increased intracellular ROS and decreased SOD activity, indicating elevated oxidative stress. CL-80 significantly reduced ROS accumulation and restored SOD levels in a concentration-dependent manner, consistent with previous reports on curcumin’s antioxidative effects [45, 47]. Although our data show that CL-80 protects against NMDA-induced neurotoxicity in cortical neurons, we did not directly assess NMDA receptor current modulation via electrophysiology or calcium imaging. Future studies using NMDA antagonists (MK-801 or D-APV) in co-treatment or antagonist-rescue designs, as well as intracellular Ca^2+^ imaging and electrophysiological recordings, are required to establish a mechanistic role of CL-80 in NMDA receptor regulation.

Experimental and clinical studies suggested that major PS-induced disorders, such as depression, may be caused at least in part by the metabolic dysregulation of monoaminergic systems in the brain [48]. Monoamine neurotransmitters in the central nervous system (CNS), particularly 5-HT, dopamine (DP), and norepinephrine (NE), are essential for regulating cognition, mood, and emotions [49]. Among them, 5-HT receptor antagonists exhibited antidepressant effects, whereas 5-HT receptor agonists produced strong anti-hyperalgesia effects [50]. The present study demonstrates that CL-80, enriched in curcumin, exhibits robust neuroprotective and antidepressant-like effects through dual modulation of NMDA and 5-HT_7A_ receptor-related pathways. While the antidepressant potential of curcumin has been previously reported in various behavioral and cellular models, prior investigations have primarily focused on classical monoaminergic systems, especially 5-HT_1A_R and 5-HT_2A_R [25, 33-35]. In contrast, receptor subtype specificity, particularly regarding 5-HT_7A_R, and glutamatergic involvement via NMDA receptor modulation have been underexplored. While individual pathways involving NMDA or serotonergic receptors have been studied, the convergent modulation of both pathways under glucocorticoid stress using a single natural extract represents a novel finding and contributes meaningfully to the neuropharmacological literature. In our study, CL-80 selectively inhibited 5-HT_7A_R-mediated cAMP signaling and ERK1/2 phosphorylation in 5-HT_7A_R-overexpressing CHO-K1 cells. A particularly novel aspect of this study is the selective inhibitory effect of CL-80 and curcumin on 5-HT_7A_R-mediated cAMP signaling. Furthermore, we investigated CL-80 inhibition of ERK1/2 phosphorylation. Treatment with 5-HT alone significantly increased ERK1/2 phosphorylation. On the other hand, SB-269970 and different CL-80 concentrations attenuated the 5-HT-induced ERK1/2 phosphorylation. This selective inhibition was also observed with curcumin and mimicked the action of SB-269970, a known 5-HT_7A_R antagonist [51, 52]. These results complement prior findings that curcumin modulates serotonergic systems, including 5-HT_1_A, _1_B, and _7_R pathways [25, 34]. 5-HT_7_R is the most recently cloned subtype of the 5-HT receptor family; its four isoforms (5-HT_7A_R, 5-HT_7B_R, 5-HT_7C_R, and 5-HT_7D_R) are all coupled to G_αs_ proteins [53-55]. Recently, 5-HT_7_R have been implicated in depression, anxiety, learning and memory deficits, sleep disorders, and neuropathic pain. Chronic antidepressant treatment has been shown to downregulate 5-HT_7_R expression, and knockout models exhibit antidepressant-like phenotypes [51, 52, 55-57]. However, only a few 5-HT_7_R antagonists have been reported [58-61]. The 5-HT_7_R antagonists DR-4004 and SB-269970 are being developed for the treatment of depression and sleep disorders [62-64]. 5-HT_7_R is a promising target for the therapy of depression and neuropathic pain. Our results suggest that CL-80 may function as a selective antagonist of 5-HT_7A_R, potentially contributing to its antidepressant efficacy. Despite the observed dose-dependent inhibition of 5-HT-induced cAMP accumulation and downstream ERK1/2 phosphorylation by both CL-80 and curcumin in 5-HT_7A_-overexpressing CHO-K1 cells, we acknowledge that these data do not establish definitive receptor-specific binding or causal *in vivo* linkage. In particular, we did not conduct radioligand binding assays to assess receptor affinity, nor did we include receptor-selective antagonists such as SB-269970 in behavioral tests. Thus, while the current results strongly suggest that CL-80 and curcumin exert antagonist-like effects on 5-HT_7A_R signaling, further validation is needed. Future studies incorporating co-administration of SB-269970 with CL-80 in the FST, or antagonist rescue experiments, will be essential to confirm the behavioral specificity of 5-HT_7A_R involvement.

Among all tested fractions, the *n*-BuOH fraction showed the highest curcuminoid content and the strongest neuroprotective and antidepressant-like activities. These observations were confirmed by TLC, HPLC, and behavioral assays. This aligns with previous phytochemical studies indicating that polar fractions, particularly *n*-BuOH, are enriched in bioactive polyphenols and flavonoids [26]. Curcumin, the major active component in this fraction, showed dose-dependent neuroprotective and serotonergic-modulatory effects, confirming its role as a pharmacodynamic marker.

In a restraint stress-induced depression mouse model, both CL-80 and curcumin significantly improved depressive-like behaviors, including reductions in immobility and increases in climbing and swimming times in the FST. The FST is a well-known behavioral examination that can assess pharmacological antidepressant activity [65, 66] with a good predictive validity. The characteristic behaviors evaluated in the FST are named “immobility” and “climbing”, after the main activities observed in animal models of depression [67, 68]. Many antidepressant drugs can reduce immobility times and increase climbing times in rodents [66]. These behavioral improvements were comparable or superior to fluoxetine, suggesting that CL-80 exerts its antidepressant actions via both monoaminergic and non-monoaminergic (glutamatergic) mechanisms. The FST is sensitive to serotonergic modulation, and increased swimming time is considered an indicator of serotonergic antidepressant activity [65]. Our results thus reinforce the relevance of 5-HT_7A_ receptor inhibition by CL-80 in mediating its antidepressant like effects. Although further tests will be required to determine the specific mechanisms of action of CL-80, our data clearly demonstrate that CL-80 has antidepressant-like effects both *in vitro* and *in vivo* in a restraint-induced stress model, suggesting that it may be an effective therapeutic option for PS-related disorders. We acknowledge several limitations of the current study despite these results. The restraint stress model employed here reflects an acute physical stress paradigm and may not fully capture the multifactorial nature of chronic human depression, which includes social, cognitive, and environmental stressors. Furthermore, the FST, used as the sole behavioral assay, though widely accepted for evaluating antidepressant-like activity, is sensitive to changes in locomotor function and does not assess other core depressive symptoms such as anhedonia or impaired cognition. To address this, future studies should incorporate complementary behavioral assays, such as the Tail Suspension Test (TST) for behavioral despair, the Sucrose Preference Test (SPT) for anhedonia, and the Open Field Test (OFT) to assess general locomotor activity and anxiety-like behaviors. In future studies, we plan to include additional behavioral tests such as TST, SPT, and OFT to validate antidepressant-like effects and rule out potential locomotor influences. In addition, the exclusive use of male mice limits the generalizability of the findings, as sex-based differences in hypothalamic-pituitary-adrenal (HPA) axis reactivity and antidepressant response have been documented [48, 49]. Nevertheless, additional study will be needed to further validate our findings and to determine CL-80 clinical efficacy and potential long-term side effects. CL-80 offers a promising natural alternative to synthetic antidepressants, addressing limitations such as delayed onset of action and side effects. However, issues such as poor bioavailability of curcumin must be addressed through formulation strategies like nanoparticle delivery or phospholipid complexes [69]. Furthermore, although behavioral and cellular efficacy was observed at the tested doses, direct measurement of curcumin plasma or brain levels was not performed. Given the low systemic bioavailability of native curcumin, future studies should incorporate PK profiling and formulation strategies to enhance translational validity.

## Conclusion

In conclusion, CL-80 exerts potent neuroprotective and antidepressant-like effects through dual modulation of the NMDA and 5-HT_7A_ receptor pathways. These effects are mediated by curcumin-enriched *n*-BuOH fractions, which reduce oxidative stress, enhance neuronal survival, and normalize serotonergic signaling. Our findings provide compelling evidence for the development of CL-80 as a novel phytotherapeutic agent for stress-related affective disorders. Future studies should focus on clinical validation, pharmacokinetics, and formulation optimization.

## Figures and Tables

**Table 1:** Extraction yield of *C. longa* under different experimental conditions.

Extracts		Cold water extract (CL-CW.)	Hot water extract (CL-HW)	20% Ethanol extract (CL-20.)	80% Ethanol extract (CL-80)
Experimental parameters	Solvent	water	water	20% Ethanol	80% Ethanol
	Temperature (°C)	25	100	90	75
	Extraction time (h)	4	4	4	4
	Solvent ratio (v/w ratio)	20	20	20	20
Extraction yield (%)		9.47	18.8	15.6	13.6

Extraction was performed using four solvent systems: cold water (CL-CW), hot water (CL-HW), 20% ethanol (CL-20), and 80% ethanol (CL-80). Each extraction was carried out for 4 h with a fixed solvent-to-solid ratio of 20 (*v/w*). The extraction temperature varied by condition, ranging from 25°C to 100°C. The resulting extraction yields (% *w/w*) were calculated based on the dry weight of extract obtained relative to the raw material weight.
